# Idebenone Protects against Acute Murine Colitis via Antioxidant and Anti-Inflammatory Mechanisms

**DOI:** 10.3390/ijms21020484

**Published:** 2020-01-12

**Authors:** Sonia Shastri, Tanvi Shinde, Sukhwinder Singh Sohal, Nuri Gueven, Rajaraman Eri

**Affiliations:** 1 Department of Laboratory Medicine, School of Health Sciences, College of Health and Medicine, University of Tasmania, Launceston, Tasmania 7250, Australia; Tanvi.Shinde@utas.edu.au (T.S.); Sukhwinder.Sohal@utas.edu.au (S.S.S.); 2 Centre for Food Safety and Innovation, Tasmanian Institute of Agriculture, University of Tasmania, Launceston, Tasmania 7250, Australia; 3 Pharmacy, School of Medicine, College of Health and Medicine, University of Tasmania, Hobart, Tasmania 7005, Australia; Nuri.Guven@utas.edu.au

**Keywords:** idebenone, cytokines, inflammatory bowel disease, lipid peroxidation, superoxide dismutase, tight junction proteins and ulcerative colitis

## Abstract

Oxidative stress is a key player of the inflammatory cascade responsible for the initiation of ulcerative colitis (UC). Although the short chain quinone idebenone is considered a potent antioxidant and a mitochondrial electron donor, emerging evidence suggests that idebenone also displays anti-inflammatory activity. This study evaluated the impact of idebenone in the widely used dextran sodium sulphate (DSS)-induced mouse model of acute colitis. Acute colitis was induced in C57BL/6J mice via continuous exposure to 2.5% DSS over 7 days. Idebenone was co-administered orally at a dose of 200 mg/kg body weight. Idebenone significantly prevented body weight loss and improved the disease activity index (DAI), colon length, and histopathological score. Consistent with its reported antioxidant function, idebenone significantly reduced the colonic levels of malondialdehyde (MDA) and nitric oxide (NO), and increased the expression of the redox factor NAD(P)H (nicotinamide adenine dinucleotide phosphate) dehydrogenase quinone-1 (NQO-1) in DSS-exposed mice. Immunohistochemistry revealed a significantly increased expression of tight junction proteins, which protect and maintain paracellular intestinal permeability. In support of an anti-inflammatory activity, idebenone significantly attenuated the elevated levels of pro-inflammatory cytokines in colon tissue. These results suggest that idebenone could represent a promising therapeutic strategy to interfere with disease pathology in UC by simultaneously inducing antioxidative and anti-inflammatory pathways.

## 1. Introduction

Inflammatory bowel disease (IBD) is characterised as a chronic, relapsing, multifactorial disorder of the gastrointestinal tract with an unknown aetiology. It is primarily divided into two major sub-forms: ulcerative colitis (UC) and Crohn’s disease (CD) [1]. IBD often presents with colonic or extra-colonic manifestations, along with clinical symptoms of abdominal pain, diarrhoea, bloody stools and weight loss. Both genetic susceptibilities and environmental factors lead to the initiation of disease [1]. However, the exact underlying mechanisms are yet to be elucidated. Many studies implicate a defective immune response, oxidative stress, altered barrier integrity and microbial dysbiosis of the gut in the pathogenesis of IBD [2]. Numerous animal models of IBD have utilised chemicals such as dextran sodium sulphate (DSS), oxazolone, acetic acid and tri-nitrobenzene sulphonic acid (TNBS) to investigate the molecular mechanisms of colitis. The potential of these preclinical models of colitis for targeted therapy have been widely described [3,4,5,6,7].

The DSS mouse model is one of the well-established indispensable tools for studying intestinal inflammation that recapitulates the morphological and clinical symptoms of human IBD, such as weight loss, diarrhoea, rectal bleeding, shortening of the colon, depletion of goblet cells and ulcerations [8,9]. DSS is thought to exert its action by irritating the epithelium, which increases the permeability of intestinal membranes by changing the expression of tight junction (TJ) proteins, such as zona-occludin 1 (ZO-1), occludin and claudins as early as day 1 of DSS administration [10,11,12]. These chronological alterations lead to a defective mucosal defence, which results in infiltration of the immune cells and release of pro-inflammatory cytokines [13,14]. Oxidative stress due to the overproduction and release of reactive oxygen species (ROS) by macrophages and other immune cells also plays a fundamental role in the pathophysiology of DSS-induced inflammation [15].

The imbalance between the levels of radicals and antioxidative enzymes contributes to oxidative stress and initiates the peroxidation of fatty acids and lipoproteins in cell membranes. The end product of lipid peroxidation (malondialdehyde) is highly toxic for cells and tissues [16]. In response to oxidative stress, the redox-sensitive transcription factor nuclear factor erythroid 2-related factor 2 (Nrf-2) initiates an antioxidant response by activating the expression of phase II detoxifying enzymes, such as NAD(P)H quinone oxidoreductase 1 (NQO-1), hemeoxygenase 1 (HO-1) and superoxide dismutase (SOD). This adaptive response is cytoprotective and enhances desensitisation against cytotoxicity and oxidative damage. There is some evidence that activation of the Nrf-2/NQO-1 redox pathway via pharmacological pre-conditioning can ameliorate DSS-induced inflammation in mice [17,18,19]. Current treatment of ulcerative colitis with non-steroidal anti-inflammatory drugs (NSAIDS), allopurinol, corticosteroids, immunosuppressant and biological agents show potential adverse effects, such as steroid dependency and infections [20,21,22,23]. Given the need to develop new therapeutic approaches to treat intestinal inflammation and oxidative damage, therapeutics that synergise antioxidant and anti-inflammatory activities could be a promising approach for the treatment of UC.

The short-chain quinone idebenone is a potent antioxidant and mitochondrial electron donor [24]. Idebenone was developed by Takeda Pharmaceuticals for the treatment of dementia in the 1980s. At present, it is marketed in Europe by Santhera Pharmaceuticals for the treatment of a rare inherited mitochondrial disorder and trials are ongoing for neurodegenerative and neuromuscular indications [25]. Idebenone is well tolerated and safe as either a single dose or multiple dose daily [26]. The related molecule MitoQ, a mitochondrial targeted derivative of idebenone, was previously reported to ameliorate DSS-induced intestinal inflammation in mice [27]. In addition, in a rat model of titanium-dioxide-induced kidney toxicity, idebenone reduced the malondialdehyde (MDA) levels; the pro-inflammatory cytokines interleukins 1, 6 and tumor necrosis factor-alpha (IL-1, IL-6 and TNF-α); and nitric oxide (NO) levels [28]. This reduction was associated with improvement in kidney function. In a model of LPS (lipopolysaccharide)-induced neuro-inflammation, idebenone alleviated the disease phenotype, which was associated with a reduction of pro-inflammatory cytokines, reduction of inducible nitric oxide synthase (iNOS) and by maintaining the polarisation between M1 and M2 macrophages [29]. However, there is only limited information about the exact mechanism(s) of action of idebenone. In vivo and in vitro evidence suggests that idebenone inhibits lipid peroxidation and maintains the redox balance by scavenging free radicals, including peroxynitrite, superoxides and peroxyls [30,31,32,33,34,35]. Furthermore, idebenone also interacts with the mitochondrial electron transport chain and maintains cellular ATP production by transferring electrons to complex 3 of the electron transport chain [36,37]. The effectiveness of idebenone depends on the two-electron reduction by the cytoplasmic flavoprotein NQO-1, which also prevents the formation of unstable cytotoxic semi-quinone radicals [38]. This reduction of idebenone limits the formation of superoxide radicals and contributes to its antioxidative activity [39,40].

We therefore hypothesised that idebenone might be a promising drug to alleviate DSS-induced inflammation due to the combination of anti-inflammatory and antioxidant activities. The current study demonstrated that idebenone successfully ameliorated intestinal inflammation and maintained tissue redox homeostasis, which was associated with an upregulation of the antioxidant enzyme NQO-1.

## 2. Results

### 2.1. Idebenone Improved the Clinical and Macroscopic Features of Colitis

**Figure 1:**
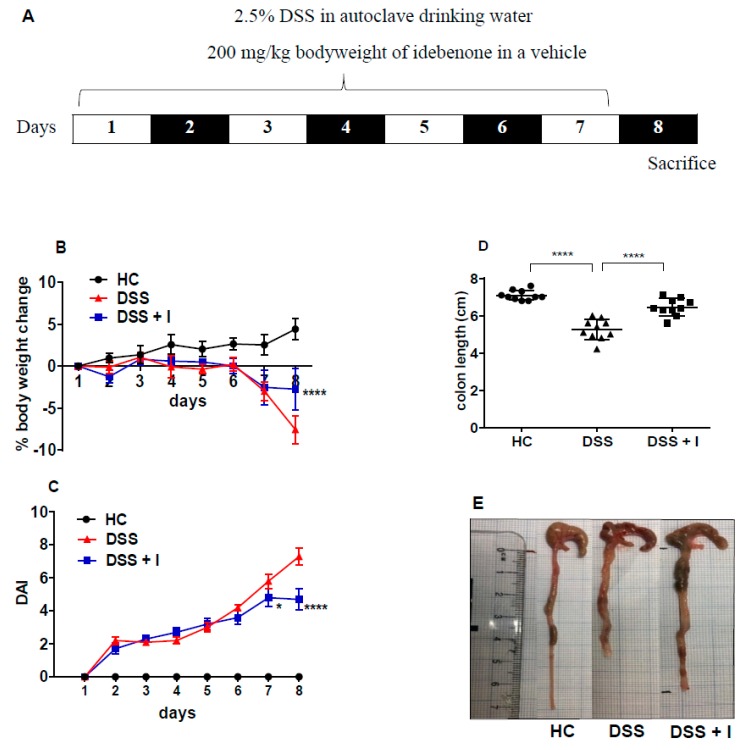
Effect of idebenone on the pathology of dextran sodium sulphate (DSS)-induced experimental colitis. (**A**) Experimental design for the administration of DSS and idebenone in C57BL/6J mice, (**B**) % body weight change, (**C**) disease activity index (DAI) of healthy controls (HC), DSS and DSS-plus-idebenone-treated mice (DSS + I). Statistical significance among the groups was evaluated using two-way ANOVA followed by Tukey’s post-test, where * *p <* 0.05 and **** *p <* 0.0001 versus DSS. Data are expressed as a mean ± SEM (n = 10/group). (**D**) Colon length and (**E**) macroscopic appearance of colon as a mean ± SEM (n = 10/group), evaluated using one-way ANOVA followed by Tukey’s post-test.

Administration of 2.5% DSS for 7 days (Figure 1A) replicated the early clinical symptoms of colitis in mice, such as body weight loss, gross rectal bleeding and diarrhoea. In comparison with healthy controls (HC) (4.48 ± 1.29%), DSS-treated animals continuously lost body weight until the end of the experiment, i.e., till day 8 (−7.56 ± 1.67%) (Figure 1B). Under these conditions, idebenone effectively prevented body weight loss (−2.74 ± 2.54%) in DSS-treated mice till day 8. The disease activity index (DAI), calculated as a composite of occult faecal blood, stool consistency and body weight, was significantly (*p* < 0.0001) increased in DSS-treated mice from day 2 onwards (Figure 1C). Idebenone treatment significantly reduced the severity of disease symptoms as evidenced by improvements in faecal blood and watery stools from day 7 (*p <* 0.05) until the end of the observation period on day 8 (*p <* 0.0001) compared to DSS-treated animals.

DSS-induced inflammation significantly (*p* < 0.0001) shortened the colon length (5.25 cm ± 0.17), in contrast with HC (7.09 cm ± 0.08). Consistent with the reduction in disease severity, idebenone treatment significantly (*p* < 0.0001) normalised the colon length compared to DSS-treated animals (6.46 cm ± 0.14) (Figure 1C,D). This data suggests a beneficial effect of idebenone treatment on the clinical symptoms of experimental acute colitis.

### 2.2. Idebenone Reduced the Colon Histopathology in Acute Colitis

**Figure 2:**
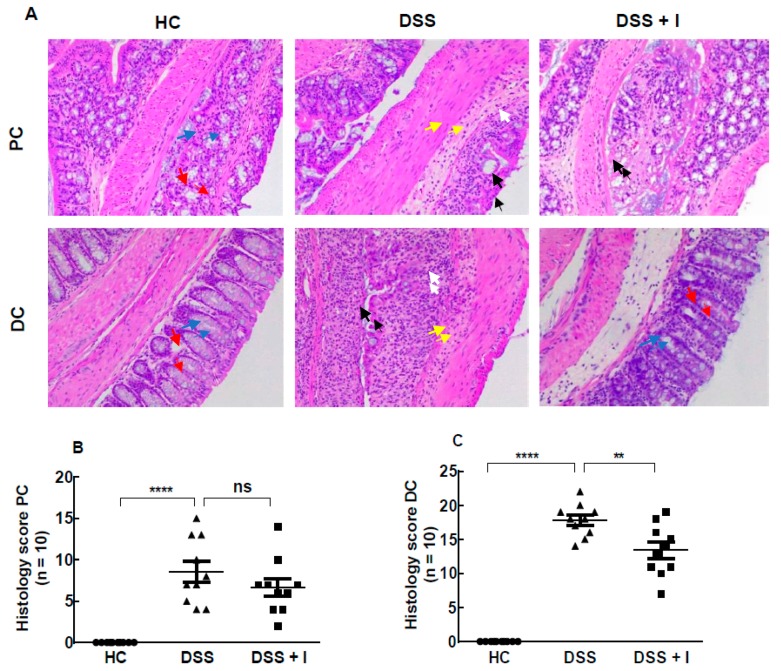
Effect of idebenone on histopathology in DSS-induced colitis. (**A**) Histological representation of proximal colon (PC) and distal colon (DC) sections stained with haematoxylin and eosin (H&E) for healthy controls (HC), DSS-treated mice (DSS) and DSS-plus-idebenone-treated mice (DSS + I) at 20× magnification. (**B**,**C**) Histopathology scores for each animal calculated after microscopic analysis of tissue sections from the PC and DC. Statistical significance among groups was evaluated using one-way ANOVA followed by Tukey’s post-test, where ns denotes non-significance, ** *p* < 0.01 and **** *p* < 0.0001. Data are expressed as a mean ± SEM (*n* = 10/group). Arrows indicate crypts/regeneration of crypts (red), goblet cells (blue), epithelium surface erosion (black), inflammatory cells infiltration (white) and submucosal oedema (yellow).

The histopathology of the proximal (PC) and distal colon (DC) was assessed using haematoxylin and eosin (H&E) staining of tissue sections (Figure 2A). Colon tissues from the HC group displayed the integrity of colonic mucosal structures without signs of inflammation. In contrast, DSS-treated mice showed severe deterioration of crypts, loss of goblet cells, annihilation of epithelial cells, submucosal oedema and massive infiltration of inflammatory cells. These DSS-induced changes were associated with higher cumulative histology scores when compared to HC (Figure 2B,C) and affected the DC (17.80 ± 0.75) more than the PC (9.40 ± 0.71) (Figure 2B). In DSS-treated mice, idebenone treatment markedly protected against colonic inflammation in the DC by preventing intestinal injury and significantly lessening the histology score (13.40 ± 1.18) (Figure 2C). Unlike its effect in the DC, idebenone treatment showed no significant protection in the PC (6.70 ± 1.06) (Figure 2B).

### 2.3. Idebenone Preserved the Intestinal Barrier Integrity and Protected against Goblet Cell Loss in DSS-Induced Colitis

**Figure 3:**
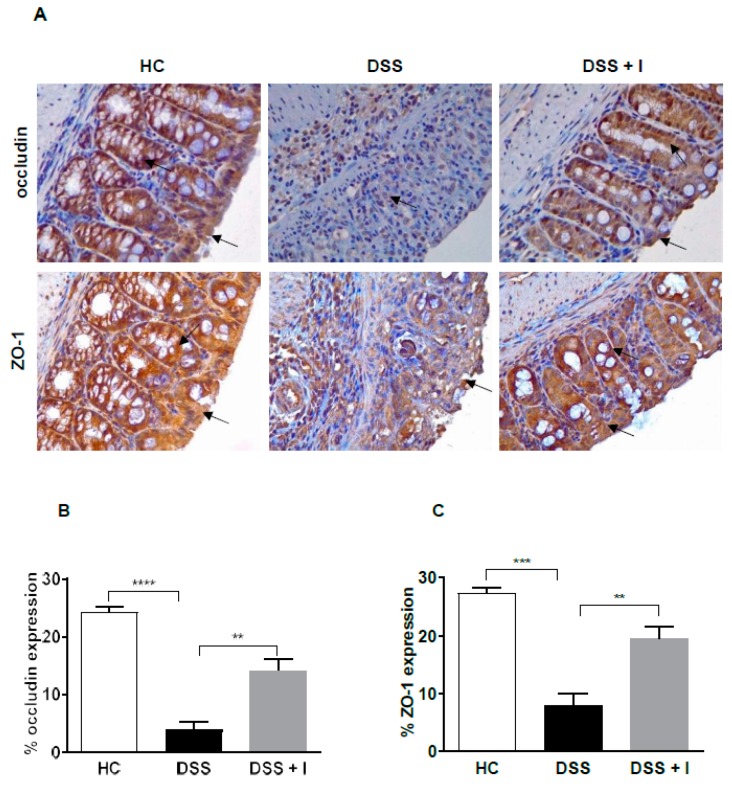
Effect of idebenone on tight junction protein expression in DSS-induced experimental colitis. (**A**) Immunohistochemical analysis of occludin and zona-occludin 1 (ZO-1), (**B**) average occludin expression in the distal colon and (**C**) average ZO-1 expression in the distal colon. Data are expressed as a mean ± SEM (n = 3/group) and statistical significance was evaluated using one-way ANOVA followed by Tukey’s post-test, where ** *p* < 0.01, ***p < 0.001 and **** *p* < 0.0001. Images are at 40× magnification. Arrow indicates the localization of staining.

Tight junction (TJ) proteins are essential for the maintenance of the intestinal epithelial barrier that limits the entry of harmful molecules into the lamina propria. Therefore, the expression of TJ proteins occludin and ZO-1 was assessed using immunohistochemistry. In HC, occludin and ZO-1 were expressed homogenously and were mainly detected around the surface membrane and around the crypts (Figure 3A) of the epithelium. However, in response to DSS, the colonic barrier integrity and crypt structure was severely affected, resulting in the low expression of TJ proteins (Figure 3A). In contrast, idebenone treatment preserved and maintained the intestinal barrier integrity, which was associated with high expression levels of occludin and ZO-1 (Figure 3B,C) along the epithelial cell membrane and intact colonic crypts (Figure 3A).

**Figure 4:**
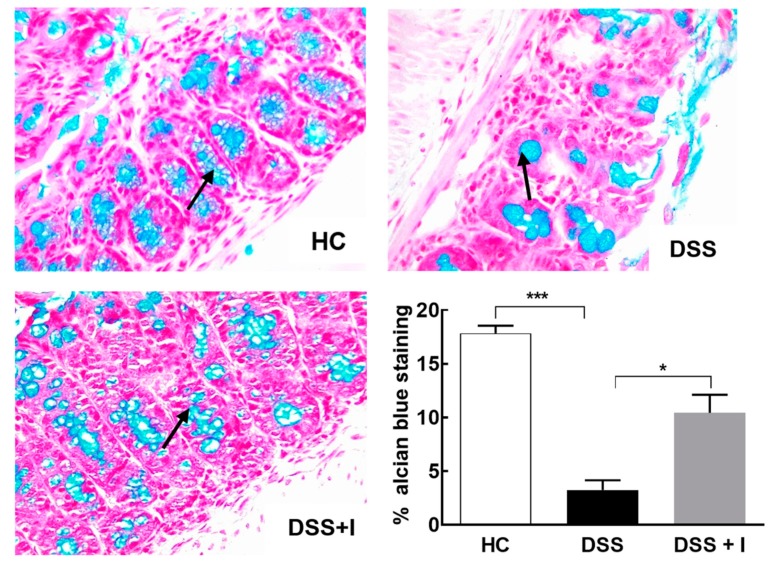
Effect of idebenone on goblet cell loss in colitis. Goblet cells producing mucus stained with alcian blue dye for HC, DSS and DSS + I groups in the distal colon, along with a graphical representation of the staining intensity of alcian blue dye for each group (*n* = 3/group). Statistical significance among groups was evaluated using one-way ANOVA followed by Tukey’s post-test, where * *p* < 0.05 and *** *p* < 0.001. Images are at 40× magnification.

Alcian blue staining was performed to quantify the mucus level in the intestinal goblet cells. Consistent with a general protective effect, idebenone significantly (*p* < 0.05) increased mucus levels compared to the reduced mucus levels in DSS-treated animals (Figure 4). 

### 2.4. Idebenone Reduced the Oxidative Stress in DSS-Induced Colitis

**Figure 5:**
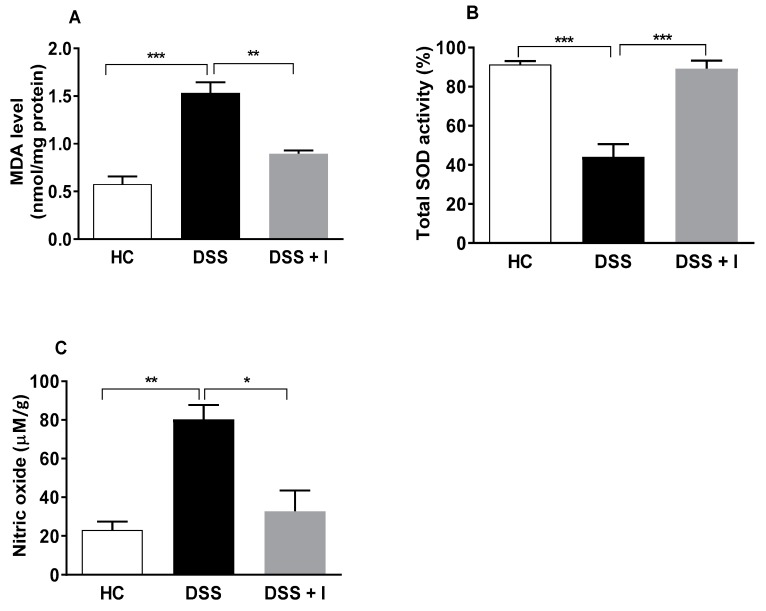
Effect of idebenone on oxidative stress in DSS-induced colitis. (**A**) Malondialdehyde (MDA) levels in the distal colon, (**B**) total superoxide dismutase (SOD) activity in the distal colon expressed as a percentage inhibition rate of reduction of xanthine oxidase activity and (**C**) nitric oxide (NO) concentration (μM)/gram in distal colon tissue. Data are expressed as a mean ± SEM (*n* = 3/group). Statistical significance among groups was evaluated using one-way ANOVA followed by Tukey’s post-test, where * *p* < 0.05, ** *p* < 0.01 and *** *p* < 0.001.

Malondialdehyde (MDA), a lipid peroxidation by-product, is one of the main indicators of oxidative damage to lipid membranes and proteins. Thus, the levels of MDA in colon tissue of the test animals were determined (Figure 5A). DSS-treated animals showed a significant increase in colon MDA levels compared to HC mice. However, in DSS-exposed mice, idebenone significantly reduced the colonic content of MDA (*p* < 0.01) in the DC (Figure 5A). We further examined the activity of the antioxidant enzyme superoxide dismutase (SOD). The DSS-treatment reduced the SOD activity by 44.13% in the DC, while under these conditions, idebenone significantly increased the SOD activity (*p* < 0.01 vs. DSS, 89.32%) to levels comparable to healthy animals (HC) (91.40%) (Figure 5B). Nitric oxide (NO), which was significantly (*p* < 0.01) increased by DSS-treatment, was significantly supressed by idebenone in the DC (*p* < 0.05, −47.49 (Figure 5C).

### 2.5. Idebenone Upregulated the Expression of the Phase II Detoxifying Enzyme NQO-1

**Figure 6:**
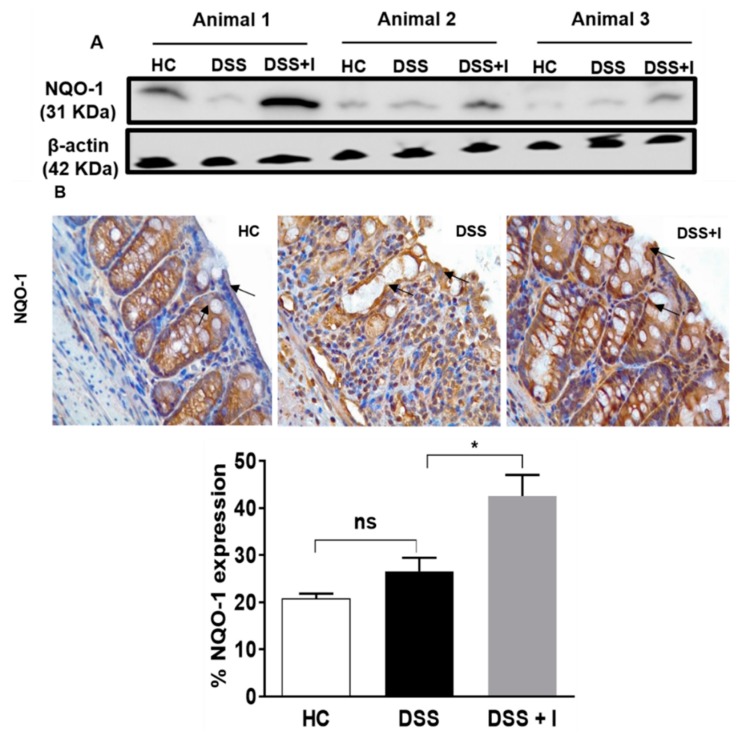
Effect of idebenone on NAD(P)H dehydrogenase quinone-1 (NQO-1) expression in colon tissue. (**A**) Protein levels of NQO-1 analysed using western blotting. (**B**) Immunohistochemical analysis of NQO-1 for all the groups, along with their graphical representation of the percentage expression in the distal colon. Data are expressed as a mean ± SEM (n = 3/group) and statistical significance was evaluated using one-way ANOVA followed by Tukey’s post-test, where ns denotes non-significance, * *p* < 0.05.Images captured using a microscope at 40×. Arrow indicates localisation of the staining.

As another readout of an Nrf-2-driven antioxidant response, the tissue levels of NQO-1 were assessed in the test animals using western blotting. While DSS exposure itself did not affect NQO-1 protein levels, idebenone up-regulated NQO-1 protein in the DSS-treated mice (Figure 6A). When the subcellular localisation of NQO-1 was assessed using immunohistochemistry, NQO-1 was mainly expressed in the cytoplasm of epithelial cells in healthy animals (Figure 6B). In contrast, in the DSS-exposed animals, only a diffuse expression of NQO-1 around the lamina propria was detected (Figure 6B). Idebenone treatment significantly increased the expression of NQO-1 in the lamina propria (*p* < 0.05 vs. DSS) (Figure 6B).

### 2.6. Idebenone Reduced the Levels of Pro-Inflammatory Cytokines in Colon Tissue

**Figure 7:**
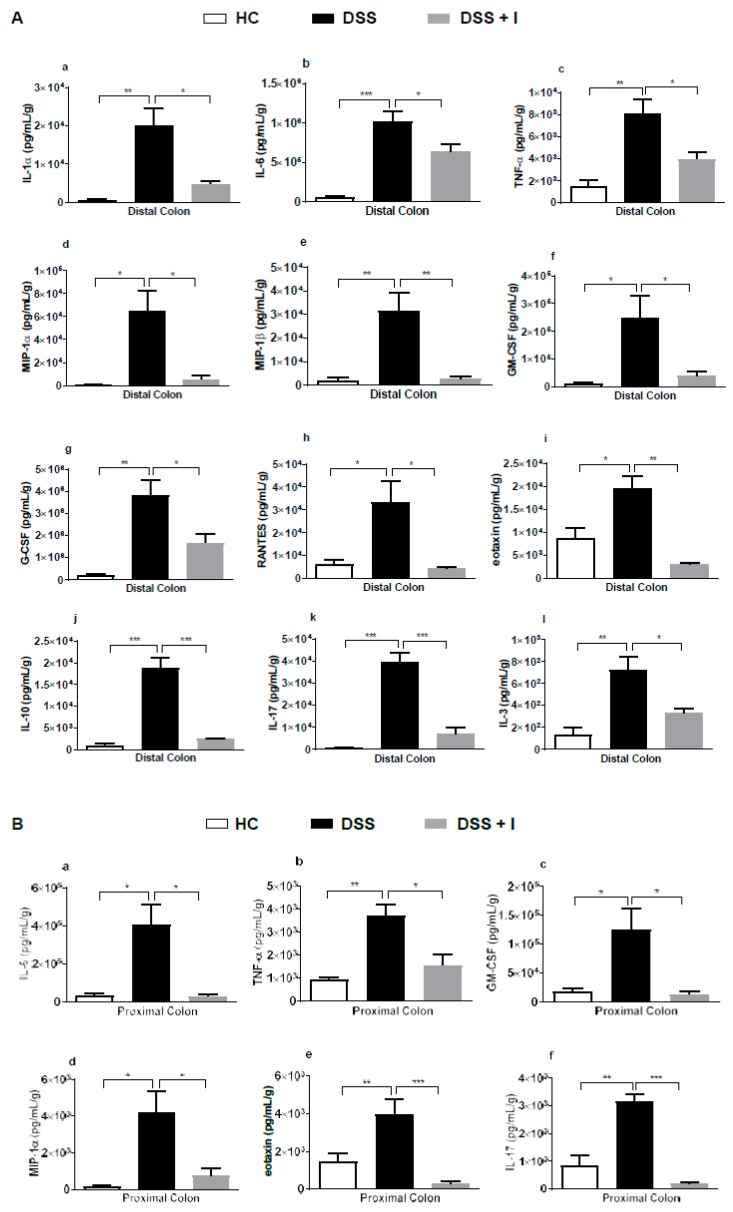
Effect of idebenone on the level of pro-inflammatory cytokines and chemokines in colon tissue. (**A**) Tissue levels of interleukin (IL)-1α, IL-6, tumour necrosis factor alpha (TNF-α), macrophage inflammatory protein 1 alpha (MIP-1α), MIP-1β, granulocyte colony stimulating factor (G-CSF), granulocyte macrophage CSF (GM-CSF), RANTES, eotaxin, IL-10, IL-17 and IL-3 in the distal colon and (**B**) the levels of IL-6, TNF-α, GM-CSF, eotaxin and IL-17 in the proximal colon were quantified using a Bio-Plex assay. Data are expressed as a mean ± SEM (n = 3/group) and statistical significance was evaluated using one-way ANOVA followed by Tukey’s post-test, where * *p* < 0.05, ** *p* < 0.01 and *** *p* < 0.001.

DSS-induced inflammation is associated with the release of an array of pro-inflammatory cytokines. To investigate the anti-inflammatory effects of idebenone, the colon tissue levels of pro-inflammatory cytokines and chemokines were quantified in the test animals. In the distal colon (DC), idebenone consistently downregulated the levels of IL-1α, IL-6, TNF-α, GM-CSF (granulocyte macrophage colony stimulating factor), G-CSF (granulocyte colony stimulating factor), IL-17, IL-10 and IL-3 (Figure 7A). In addition, idebenone also significantly lowered the levels of chemokines MIP-1α (macrophage inflammatory protein 1 alpha), MIP-1β (macrophage inflammatory protein 1beta), RANTES (Regulated on Activation, Normal T Expressed and Secreted) and eotaxin in the DC (Figure 7A). In the proximal colon (PC), idebenone significantly reduced IL-6, TNF-α, GM-CSF, MIP-1α, IL-10 and IL-17 in the PC (Figure 7B). Thus, in line with its protective activity, the immunomodulatory effects of idebenone were more prominent in the DC compared to the PC. No significant effects were detected for several other cytokines (Supplementary Figure S1).

## 3. Discussion

For the first time, the current study revealed a protective role for idebenone in a well-described and frequently used pre-clinical murine model of acute colitis. Our results demonstrate the substantial therapeutic potential of idebenone in reducing the severity of DSS-induced disease by simultaneously exerting both antioxidant and anti-inflammatory activities, while concomitantly regulating the immune responses and barrier function.

The current study used idebenone at a dose of 200 mg/kg that previously ameliorated oxidative stress, pro-inflammatory cytokines and DNA damage in a titanium-dioxide-induced hepatotoxicity model [41], and also improved cardiac function [42]. Since administration of idebenone with food is known to increase its bioavailability by 5 to 7 times [43], idebenone was administered with chow pellets in the present study. It must be acknowledged that the present study did not approach the question of finding an optimal drug dose, which should be included in future studies. However, based on the Food and Drug Administration (FDA)-approved conversion factor between mouse and men [44], 200 mg/kg/day in mice would translate to a dose of less than 1.2 g/day (based on a 70 kg patient body weight). Idebenone at doses of up to 2250 mg/day are considered safe and tolerable for human administration [26], which places the dose used in the current study into a possible therapeutic range that could be used in clinical trials. Although high idebenone doses can cause gastro-intestinal-related adverse effects, such as vomiting, dyspepsia and mild-to-moderate diarrhoea in patients [45], the present study did not observe any gastro-intestinal-related adverse effects. Instead, idebenone significantly improved the body weight, DAI and histopathology. Our results support the previously reported involvement of oxidative stress in the pathogenesis of disease [15] and justify the use of antioxidants to alleviate structural tissue damage [19,27,46,47].

It is well established that reactive oxygen species and reactive nitrogen species (RNS) at low concentrations and over short time intervals are essential signalling molecules involved in a multitude of physiological processes [48]. However, when cells are exposed to elevated levels of ROS and the subsequent lipid peroxidation over longer time periods, they are unable to cope with the oxidative damage that can disrupt cell and tissue functions in the gastrointestinal tract [16]. It is therefore thought that strengthening the antioxidant defence machinery counteracts the harmful effects of ROS in the body. In this context, one of the major events in the cellular response to oxidative stress is mediated by the activation of phase II detoxifying enzymes NQO-1 and SOD [49]. The antioxidants SOD and NQO-1 directly eliminate free superoxide radicals to protect against lipid peroxidation [50,51]. Previous reports of acute gut inflammation in the DSS model observed a significant increase in lipid peroxidation and reduced levels of antioxidant enzymes (NQO-1 and SOD) [18,19,52,53,54,55,56], which were replicated in the present study. We also observed a significant reduction in MDA levels after treatment with idebenone, which is consistent with a previous report where idebenone effectively suppressed lipid peroxidation in brain mitochondria [57]. In line with the activation of an endogenous defence mechanism, idebenone increased the NQO-1 expression and SOD activity rate in our study (Figure 6). Previous reports associated elevated levels of NO with the severity of colitis in UC patients and in animal models [58,59,60]. In line with idebenone-dependent protection, idebenone also influenced RNS by reducing NO levels in our study. These results indicate that idebenone protects mucosal injury by strengthening the antioxidant defence machinery.

However, it must be noted that idebenone has also been associated with a pro-oxidative activity in some reports [61], which appears counterintuitive given the abundance of its antioxidant effects. Idebenone, like all quinones, can be reduced by a single electron reduction to the unstable semiquinone, which can give rise to oxidative radicals [62]. This reaction is effectively suppressed by NQO-1 through a competing two-electron reduction to the hydroquinone. Therefore, cells and tissues with low or absent NQO-1 levels are at risk of semiquinone-induced oxidative stress. It is therefore possible that low levels of semiquinones could trigger increased NQO-1 expression in an Nrf2-dependent manner to counteract quinone-induced oxygen radicals until at higher NQO-1 concentrations, where idebenone is fully reduced to the hydroquinone to exert its antioxidant activity. More detailed studies will be required to investigate this theoretical possibility. 

The accumulation of ROS and RNS also disrupt the intestinal barrier integrity by redistributing TJ protein complexes involving occludin and ZO-1, which leads to a vicious cycle of intestinal inflammation [63,64,65,66,67,68,69,70]. Therefore, restoring barrier integrity to mediate the resolution of pro-inflammatory responses could be a beneficial strategy in the treatment of UC. In this regard, idebenone was previously reported to decrease the permeability of the blood–brain barrier by up-regulating occludin and ZO-1 proteins in a rat model of diabetes [71]. Likewise, our results in the colitis model also suggest that idebenone protected the expression of TJ proteins and preserved the integrity of the epithelial barrier. The substantial loss of TJ proteins in the DSS-treated mice in the present study mirrored previous reports [72,73]. However, it is unclear whether idebenone increased the expression of the TJ proteins or protected against their DSS-dependent degradation. Moreover, the present study also demonstrated a significant protection of goblet cells by idebenone. The loss of goblet cells upon DSS induction replicates the depletion of goblet cells in UC patients and rodents [72,74]. Indeed, this is the first study that shows a protective effect by idebenone against intestinal goblet cell loss. However, to understand the detailed molecular mechanism(s) for this effect, further detailed investigations will be required.

Gut inflammation is characterised by the overproduction of pro-inflammatory cytokines and chemokines, along with altered barrier integrity and an oxidative response [14,16,69,75]. Activation of neutrophils and macrophages in the lamina propria during mucosal injury result in inflammation via the aberrant secretion of cascades of pro-inflammatory cytokines [76]. DSS-induced acute colitis is a macrophage/Th1/Th17 driven inflammatory mouse model with elevated levels of cytokines including TNF-α (a hallmark of DSS-induced inflammation), IL-6, IL-1β and IL-17 [77,78,79,80]. Therefore, suppression of pro-inflammatory cytokines is thought to represent an essential part of the therapeutic approach against UC. Evidence for idebenone-induced anti-inflammatory activity was previously proposed in a rat model of titanium-dioxide-induced renal damage and in LPS-induced neuro-inflammation in BV2 microglial cells that involved the suppression of IL-1β, TNF-α and IL-6 [28,29]. Our results illustrate a substantial suppression of the pro-inflammatory cytokines IL-1α, TNF-α, IL-6 and IL-17 to their baseline levels by idebenone (Figure 7). In addition, idebenone also reduced the levels of GM-CSF, G-CSF and IL-3, which are responsible for the differentiation and recruitment of monocytes to macrophages into the lamina propria during intestinal inflammation [81].

It is well known that the anti-inflammatory cytokine IL-10 is elevated in colitis patients [47,82,83,84,85,86] and our study mirrored this effect upon DSS exposure. Elevated levels of IL-10 could be interpreted as a compensatory mechanism to counteract the substantial inflammation in the colon. In the context of a reduced pro-inflammatory response by idebenone, a reduction of IL-10 levels in the present study does therefore not appear surprising.

Our results also align with clinical studies that reported up-regulated chemokines, such as MIP-1α, MIP-1β, eotaxin and RANTES, in the colonic biopsies of UC patients [49,83,87,88,89]. However, profound effects were seen in our study where idebenone down-regulated the release of these chemokines in the DSS-treated mice. The significant reduction of pro-inflammatory cytokine levels by idebenone could be either a direct immunomodulatory effect or indirectly reflective of a general protection of antioxidant enzymes and/or barrier integrity. Since macrophages seem to play a vital role in colitis through the production of many cytokines [81,90], detailed studies will be required to elucidate the effect of idebenone on macrophages and to identify the functional links between ROS and cytokine levels.

Apart from DC, idebenone administration also showed significant reductions of IL-6, TNF-α, GM-CSF, MIP-1α, eotaxin and IL-17 in the PC. Our results also indicated that the inflammation level varied between PC and DC. This has been substantiated by previous studies [49,72,83], where the DC of DSS controls showed more histological destructions than the PC. 

**Figure 8:**
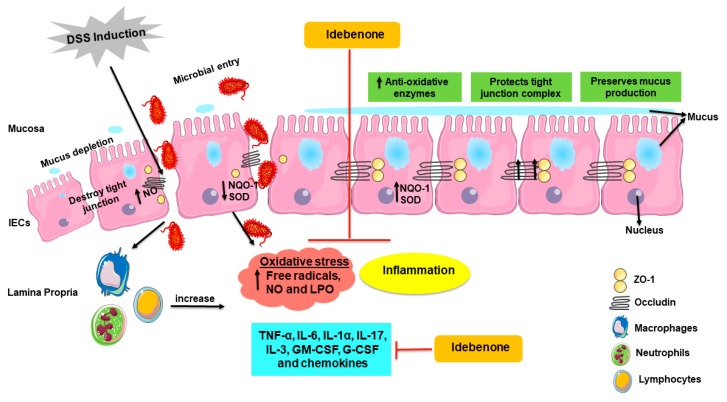
Schematic illustration of the proposed mode of action of idebenone in a mouse model of DSS-induced acute colitis. Induction of DSS disrupts tight junctions (ZO-1 and occludin) and the mucus film covering epithelial cells, resulting in the increased infiltration of harmful microbes and toxins into the lamina propria. This uptake activates macrophages, neutrophils and lymphocytes, causing dissemination of pro-inflammatory cytokines (IL-6, IL-1a, TNF-a, IL-17, IL-3, GM-CSF and G-CSF), chemokines and generates oxidative and nitrosative stress (free radicals and NO). In colitis, NO is released by immune cells, as well as by IECs, which further damages tight junctions. All these factors contribute to the inflammation of the colon. Increased levels of oxidative stress via altered redox levels between oxidative molecules and anti-oxidative enzymes (NQO-1 and SOD) damages tissue and cells through oxidative damage to macromolecules, including lipids. Supplementation with idebenone maintains the barrier integrity by protecting tight junctions and the mucin layer. Idebenone also supresses the pro-inflammatory cytokines, chemokines, NO production and LPO. In addition, by increasing the levels of detoxifying enzyme NQO-1 and SOD, idebenone is thought to prevent colonic inflammation by simultaneously protecting against oxidative stress and inflammation. IECs—intestinal epithelial cells, G-CSF—granulocyte colony stimulating factor, GM-CSF—granulocyte macrophage colony stimulating factor, IL—interleukin, LPO—lipid peroxidation, NO—nitric oxide, NQO-1—NAD(P)H dehydrogenase quinone 1, SOD—superoxide dismutase, TNF-α—tumor necrosis factor alpha and ZO-1—zona occludin 1.

Very few randomised controlled clinical trials have reported therapeutic effects of antioxidants in UC patients. Trials that tested compounds, such as curcumin and polyunsaturated fatty acids (PUFAs), were associated with methodical limitations, including short duration, small subject size or the absence of a healthy control group [91,92]. Other studies that investigated compounds such as resveratrol, carnosine and ferrous fumarate were insufficiently sized to show any therapeutic benefits. Moreover, some antioxidants showed better efficacy when given in combination with other compounds, and mixed treatment outcomes were reported as being influenced by the antioxidant potency, dosage, stage of disease and duration [93,94,95,96,97]. Therefore, at present, it is not possible to align the multiple contradicting results of the reported clinical trials. On the other side, a plethora of literature supports the role of oxidative stress in UC and consequently justifies the use of antioxidants as a therapeutic strategy [15,27,52,54,60,98,99,100,101,102]. Since UC is a multifactorial disease, pharmacological targeting of individual factors appears of limited use to provide effective and sustainable therapy outcomes. In contrast, the present study highlighted that idebenone appeared to target not only one, but multiple factors relevant to UC that included the barrier integrity, mucus production, oxidative stress and inflammatory markers (Figure 8). It is intriguing to speculate that it was the synergistic effect of these activities that could be responsible for the significantly reduced severity of colitis observed in this study.

Accumulation of oxidative stress not only damages the colon, but also stimulates the release of pro-inflammatory cytokines and increases intestinal permeability. It must be noted that at present, it is still unclear how the different idebenone-dependent events that protect against colitis are initiated and regulated, and whether there is a functional or temporal hierarchy between the different activities described in the current study. To our knowledge, the present study is the first to explore the antioxidative and anti-inflammatory properties of idebenone in attenuating the pathology of acute colitis. Idebenone provides effective cytoprotection against oxidative damage, likely by a SOD/NQO-1-dependent mechanism, maintaining intestinal barrier function and simultaneously preventing the up-regulation of pro-inflammatory cytokines in a mouse model of DSS-induced acute colitis. Therefore, our study strongly suggests that idebenone could be developed as a promising therapeutic alternative to treat the acute phases of UC.

## 4. Material and Methods

### 4.1. Animals

All female C57BL/6J mice were purchased from the University of Tasmania animal breeding facility. Mice were caged individually with access to standard chow and autoclaved drinking water *ad libitum*. Animals were housed at a controlled temperature with 12-h day/night light cycle. Individual body weights were assessed daily over an acclimatisation period of 7 days before being included into any experiments. All procedures were approved by the Animal Ethics Committee of University of Tasmania (ethics approval number: A0016166 and approval date:6^th^ March 2017. Experiments were conducted according to the Australian code of practise for the care and use of animals for scientific purposes (8th edition 2013).

### 4.2. Experimental Design and Drug Treatment

Female C57BL/6J mice aged 6–7 weeks with an average weight around 18 g were randomly divided into three groups (*n* = 10/group): healthy controls without DSS and drug (HC), DSS treatment (DSS) and DSS + idebenone treatment (DSS + I). All animals were acclimatised for 1 week before the start of the experiment. Colitis was induced by administering 2.5% DSS (molecular weight – 30,000-50,000 KDa) (MP Biomedicals, New South Wales, Australia) in autoclaved drinking water to all groups except HC, continuously over 7 days. Idebenone was prepared as wet food mash by mixing it with 0.5% of carboxymethylcellulose (CMC), 4% sucrose and autoclaved powdered chow pellets. Idebenone was administered orally at a dose of 200 mg/kg of body weight. HC were supplied with normal autoclaved water and standard autoclaved chow pellets in the form of food mash. DSS-treated and DSS + idebenone groups were treated with a drug vehicle and idebenone (200 mg/kg of body weight), respectively, for the treatment period of 7 days. Food mash was aliquoted as 2.5 g per dish and was stored at −20 °C until use.

### 4.3. Clinical and Histopathological Evaluations 

The disease activity index (DAI) was calculated as the sum of the individual scores for bloody stool, stool consistency and body weight loss, as previously described [72,103]. All three parameters were recorded daily over 7 days until the end of the experiment on day 8. In brief, scores were determined as follows: stool consistency (0 = normal, 1 = semi-formed, 2 = very soft/loose stool, 3 = diarrhoea or watery stool), bloody stool (0 = no blood/negative haemoccult, 1 = positive haemoccult, 2 = visible blood traces, 3 = gross bleeding) and body weight loss (0 = 0%, 1 = 1–5%, 2 = 6–10%, 3 = 11–15%). After dissecting the mice on day 8, the colons were taken out and the lengths were recorded. The colon was opened and longitudinally cut into two halves. One-half was collected for histopathological evaluations using the Swiss roll technique and another half was snap-frozen for further molecular assays. The Swiss roll was then fixed in 10% neutral formalin buffer and embedded in paraffin. Paraffin-embedded tissue slides were stained with haematoxylin and eosin (H&E) staining and histopathological scoring was done in a blinded manner, as described previously [104]. Images were captured using a Leica DM500 microscope (Leica Microsystems, Mannheim, Germany).

### 4.4. Immunohistochemistry

An HRP/DAB detection IHC kit from Abcam (ab64261, Abcam, Victoria, Australia) was used to perform immunohistochemical analysis, as previously described [49]. Briefly, paraffin-embedded tissues were sectioned into 5-μm slices, were dewaxed in xylene and then rehydrated in a series of graded ethanol (100%, 100%, 95% and 70%). Slides were then incubated for antigen retrieval at 121 °C for 4 min using a citrate buffer (pH 6) in a decloaking chamber. After washing in phosphate buffered saline (1× PBS), slides were incubated with a hydrogen peroxide block in order to block endogenous peroxidase activity for 10 min, followed by protein blocking for 30 min at room temperature. Subsequently, slides were incubated overnight at 4 °C with primary antibodies against occludin (1:600) (NBP1-87402, Novus Biologicals, Victoria, Australia), ZO-1 (1:400) (NBP1-85046, Novus Biologicals) and NQO-1 (1:400) (ab34173, Abcam). After washing the slides in PBS, slides were incubated with biotinylated goat anti-rabbit IgG and Streptavidin-peroxidase conjugate for 10 min each according to the manufacturer’s instructions (Abcam). Finally, slides were incubated with DAB chromogen (3,3′-diaminobenzidine) and substrate for 10 min. Tissues were counterstained using haematoxylin before being mounted with DPX medium (Sigma-Aldrich, New South Wales, Australia). Images were captured using a Leica DM500 microscope and Image Pro-Plus 7 software (Media Cybernetics, Inc., Rockville, MD, USA) to analyse the staining intensity by randomly choosing four different fields per slide (n = 3/group), with the observer being blinded to the diagnosis.

### 4.5. Western Blotting

Briefly, distal colon tissue sections were homogenised and lysed in a RIPA buffer containing protease inhibitor cocktails (Complete ULTRA Tablets, Mini, EDTA-free, Roche, New South wales, Australia). Protein quantification was done using a DC protein Assay Kit from Biorad. The samples were suspended in loading dye and boiled at 95 °C for 5 min. A total of 20 μg of protein was separated on 4–15% of SDS-PAGE gel (Mini-PROTEAN TGX Precast Gels (50 µL), Biorad, New South Wales, Australia) at 100 V for 60 min, then electro-transferred to a PVDF membrane at 250 A for 60 min. The membranes were blocked with 5% non-fat milk for 1 h at room temperature (RT) and then incubated with primary antibodies against NQO-1 (1:1000) (ab34173, Abcam) and β-actin (1:8000) (NB600-503, Novus Biologicals) overnight at 4 °C. After washing, membranes were incubated with horseradish peroxidase (HRP)-conjugated secondary antibody (1:3000, 7074, Cell Signaling Technology, Australia) for 1 h at RT. The bands were visualised with a chemiluminescence reagent (SuperSignal, West Pico PLUS, Chemiluminescent Substrate, Thermo Scientific, Victoria, Australia) and imaged using a Fujifilm Luminescent Image Analyzer (LAS-3000 image reader, version 2.2) (Fuji Life Sciences, Japan).

### 4.6. Alcian Blue Staining

Alcian blue staining kit (ab150662 Alcian Blue stain kit, pH 2.5 (Mucin Stain), Abcam, Australia) was utilised to visualise the sulphated and acidic mucopolysaccharides (MUC2). Staining was performed as described previously [105]. Briefly, paraffin-embedded slides were dewaxed in xylene and rehydrated in a series of graded ethanol. Slides were incubated with alcian blue for 30 min at RT. Slides were counterstained with Safranin O for 5 min, dehydrated and cleared in xylene before mounting with DPX medium. Images were captured using a Leica DM500 microscope and Image Pro-Plus 7 software (Media Cybernetics, Inc., Rockville, Maryland, United States of America) was used to analyse the staining intensity by randomly choosing four different fields per slide (n = 3/group), with the observer being blinded to the diagnosis.

### 4.7. Lipid Peroxidation Assay

The levels of MDA as a marker of lipid peroxidation was determined by using a commercially available lipid peroxidation colorimetric/fluorometric assay kit (K739, Bio Vision, New South Wales, Australia), as mentioned previously [106]. Briefly, the distal colon tissue was homogenised with a lysis buffer and centrifuged at 13,000× *g* for 10 min. The resulting supernatants were supplemented with thiobarbituric acid (TBA) and were boiled at 95 °C in a water bath for 60 min. The MDA-TBA adduct was formed, which was quantified colorimetrically at 532 nm. The amount of MDA in the samples was detected by plotting against an MDA standard (provided in the kit) calibration curve. The values were expressed as nmol/mg protein.

### 4.8. Measurement of SOD Activity and NO Production

Total superoxide dismutase (SOD) activity was measured using a commercially available superoxide dismutase activity assay kit (ab65354, Abcam), as described previously [107]. Briefly, distal colon tissues were homogenised in ice-cold Tris/HCl (0.1 M, pH 7.4) containing Triton X-100 (0.5%), β-mercaptoethanol (5 mM) and PMSF (phenylmethylsulfonyl fluoride) (0.1 mg/mL). After centrifugation at 14,000× *g* for 5 min, supernatants (containing cytosolic and mitochondrial SOD enzyme) were assayed by adding WST-1 (water soluble tetrazolium-1) solution according to the manufacturer’s instructions. The data represents the percentage inhibition of superoxide production by SOD and was termed the SOD activity. The more SOD in the samples, more its inhibitory activity.

For NO generation, a Griess reagent kit (G2930, Promega, Victoria, Australia) was used to measure nitrite, a stable by-product of NO, as described previously [108]. Colon tissue explants and nitrite standards (100, 50, 25, 12.5, 6.25, 3.13, 1.56 and 0 µM) were pipetted into a 96-well plate. The samples and standards were incubated according to the manufacturer’s instructions. The detection was based on the chemical reaction between sulphanilamide and *N*-1-napthylethylenediamine dihydrochloride (NED) under acidic conditions. The sample absorbance was plotted against a nitrite standard reference curve at 550 nm. The values were expressed as a concentration in μM/gram of tissue. 

### 4.9. Cytokine Measurement from the Tissue Explant Culture

Proximal and distal colon samples were excised from the test animals, washed with PBS and cultured in 12–well plates containing 500 µL/well of RPMI1640 (In Vitro Technologies Pty Ltd., Melbourne, Australia), supplemented with 10% Fetal Bovine Serum (Gibco, Life Technologies Pty Ltd., Melbourne, Australia) and 1% antibiotics solution (containing 10 mg/mL streptomycin and 10,000 U/mL of penicillin; Sigma-Aldrich Pty Ltd., New south Wales, Australia), as mentioned previously [72]. After incubating for 24 h, the supernatant was collected, centrifuged and analysed for cytokine detection. The concentration of cytokines in the colonic tissue explant were measured using a Bio-Plex Pro Mouse cytokine 23-plex kit (#M60009RDPD, Bio-Rad Laboratories, New South Wales, Australia) following the manufacturer’s protocol in a Bio-Plex 200 instrument (Bio-Rad Laboratories), and were analysed using the Bioplex Manager software, version 6 (Bio-Rad Laboratories). The cytokine levels were normalised to the measured tissue weight (gram). The concentration of cytokines was presented as pg/mL/g of tissue.

### 4.10. Statistical Analysis

GraphPad Prism software version 6.0 (GraphPad Software Ltd, La Jolla, California, United States of America) was used to perform statistical analysis. Results were expressed as a mean ± SEM from at least 3 to 10 animals per group. Statistical differences between the groups were evaluated using one-way analysis of variance (ANOVA) followed by Tukey’s post-hoc. Two-way ANOVA followed by Tukey’s post-hoc test was used to analyse DAI and body weight changes during the experimental period. A statistical difference of *p* < 0.05 was considered significant.

## Supplementary Materials

The following are available online at https://www.mdpi.com/1422-0067/21/2/484/s1, Figure S1: Effect of idebenone on the level of pro-inflammatory cytokines and chemokines in colon tissue. (A) Tissue levels of IL-1β, INF-γ, IL-12p40, IL-12p70, MCP-1, IL-2, IL-5, IL-9 and IL-13 in the distal colon and (B) the levels of IL-1α, IL-1β, INF-γ, G-CSF, MIP-1β, RANTES, IL-3, IL-12p40, IL-12p70, IL-10, IL-4 and IL-13 in proximal colon were quantified using a Bio-Plex assay. Data are expressed as a mean ± SEM (n = 3/group) and statistical significance was evaluated using one-way ANOVA followed by Tukey’s post-test, where * p < 0.05.
