# Supplementary material for: Idebenone Protects against Acute Murine Colitis via Antioxidant and Anti-Inflammatory Mechanisms
Source: Int J Mol Sci. 2020 Jan 12;21(2):484. doi: 10.3390/ijms21020484 (PMC7013829; doi:10.3390/ijms21020484)
Supplement: Supplementary file 1 [file ijms-21-00484-s001.pdf]

A

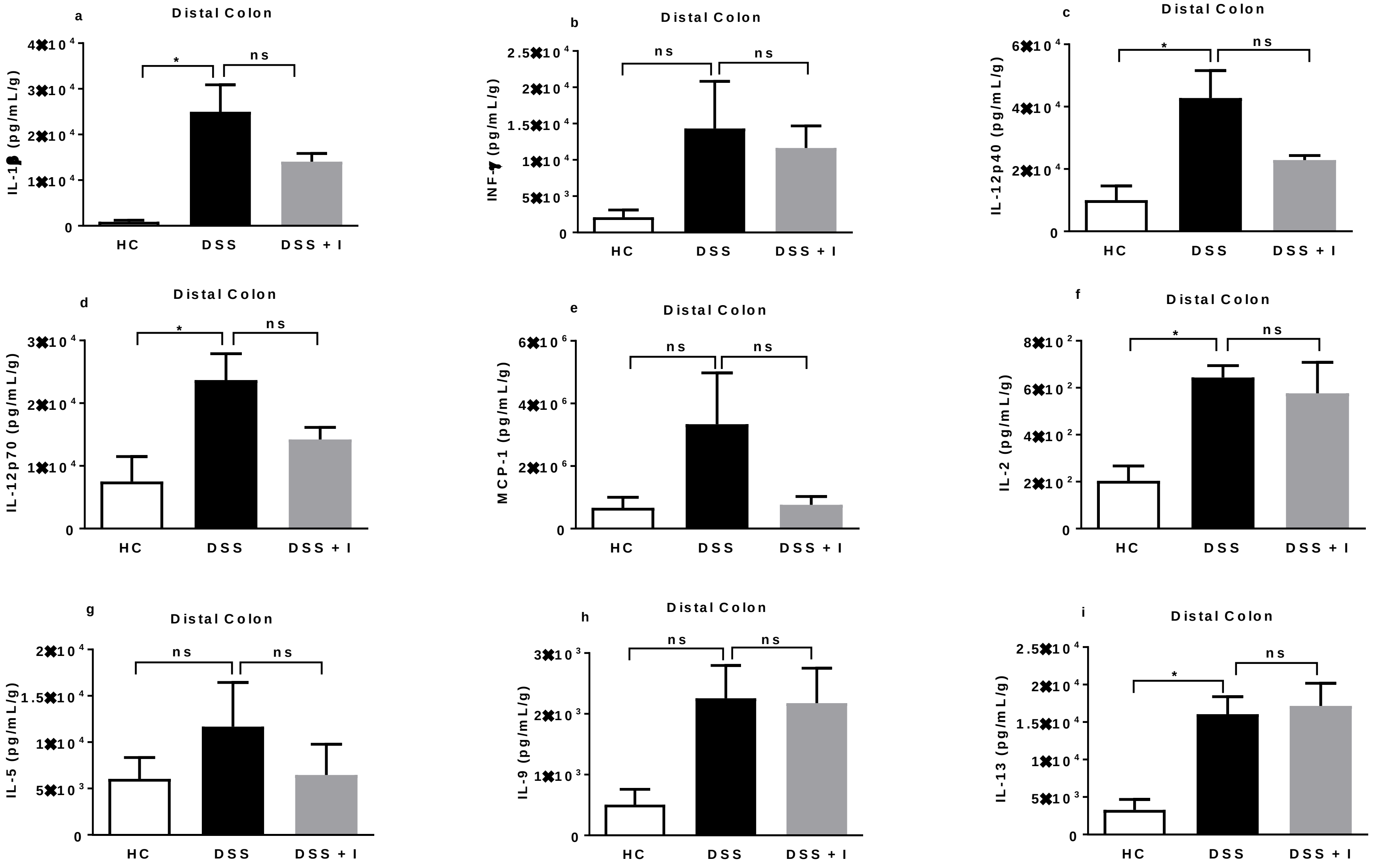

a
Distal Colon
IL-1β (pg/mL/g)
4×10^4
3×10^4
2×10^4
1×10^4
0
*
ns
HC
DSS
DSS + I
b
Distal Colon
INF-γ (pg/mL/g)
2.5×10^4
2×10^4
1.5×10^4
1×10^4
5×10^3
0
ns
ns
HC
DSS
DSS + I
c
Distal Colon
IL-12p40 (pg/mL/g)
6×10^4
4×10^4
2×10^4
0
*
ns
HC
DSS
DSS + I
d
Distal Colon
IL-12p70 (pg/mL/g)
3×10^4
2×10^4
1×10^4
0
*
ns
HC
DSS
DSS + I
e
Distal Colon
MCP-1 (pg/mL/g)
6×10^6
4×10^6
2×10^6
0
ns
ns
HC
DSS
DSS + I
f
Distal Colon
IL-2 (pg/mL/g)
8×10^2
6×10^2
4×10^2
2×10^2
0
*
ns
HC
DSS
DSS + I
g
Distal Colon
IL-5 (pg/mL/g)
2×10^4
1.5×10^4
1×10^4
5×10^3
0
ns
ns
HC
DSS
DSS + I
h
Distal Colon
IL-9 (pg/mL/g)
3×10^3
2×10^3
1×10^3
0
ns
ns
HC
DSS
DSS + I
i
Distal Colon
IL-13 (pg/mL/g)
2.5×10^4
2×10^4
1.5×10^4
1×10^4
5×10^3
0
*
ns
HC
DSS
DSS + I

B

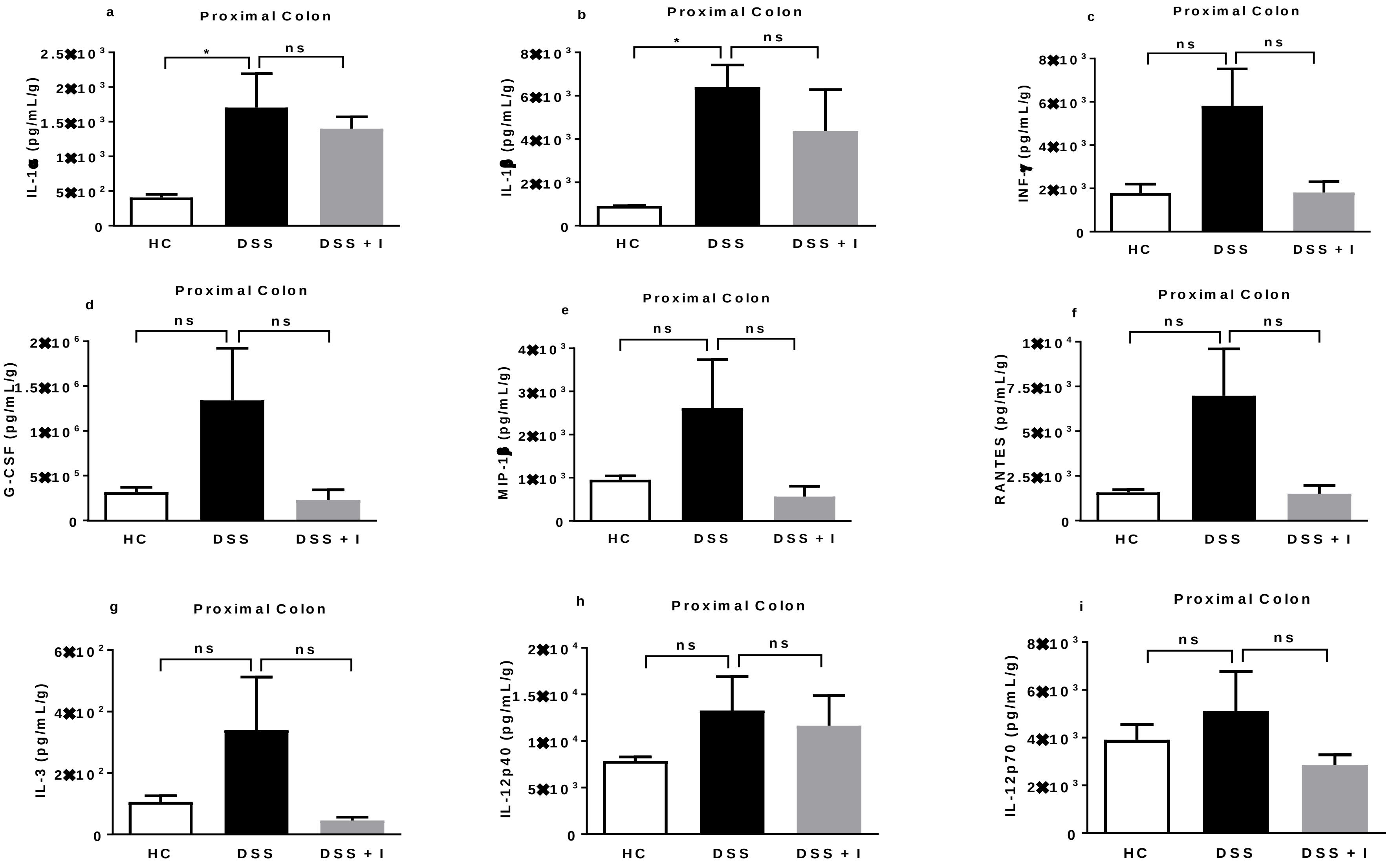

a
Proximal Colon
IL-1α (pg/mL/g)
HC
DSS
DSS + I
b
Proximal Colon
IL-1β (pg/mL/g)
c
Proximal Colon
INF-γ (pg/mL/g)
d
Proximal Colon
G-CSF (pg/mL/g)
e
Proximal Colon
MIP-1β (pg/mL/g)
f
Proximal Colon
RANTES (pg/mL/g)
g
Proximal Colon
IL-3 (pg/mL/g)
h
Proximal Colon
IL-12p40 (pg/mL/g)
i
Proximal Colon
IL-12p70 (pg/mL/g)

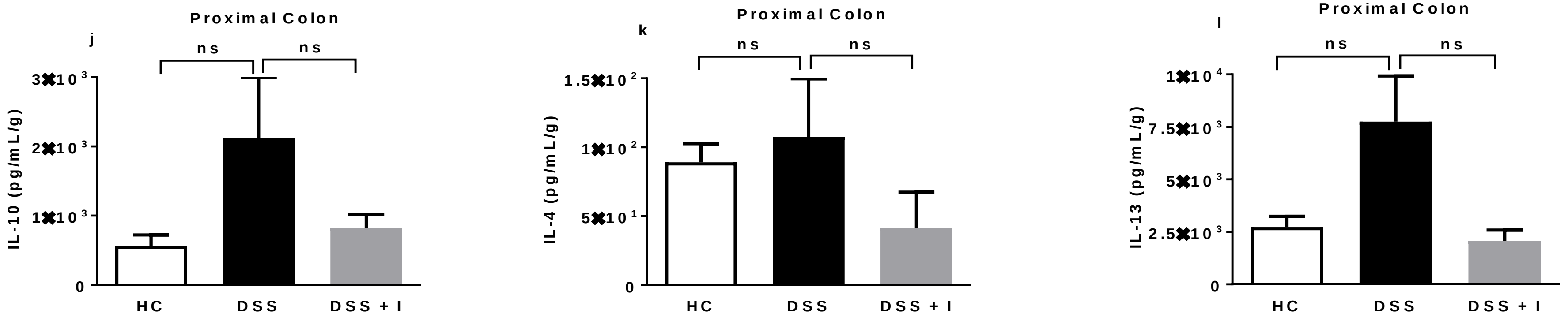

Supplementary Figure S1. Effect of idebenone on the level of pro-inflammatory cytokines and chemokines in colon tissue. **A**) Tissue levels of IL-1β, INF-γ, IL-12p40, IL-12p70, MCP-1, IL-2, IL-5, IL-9 and IL-13 in distal colon and **B**) the levels of IL-1α, IL-1β, INF-γ, G-CSF, MIP-1β, RANTES, IL-3, IL-12p40, IL-12p70, IL-10, IL-4 and IL-13 in proximal colon were quantified by Bio-Plex assay. Data expressed as mean ± SEM (n=3/group) and statistical significance evaluated by One-way ANOVA followed by Tukey's post-test where *$p<0.05$.
